# 4-(3-Methyl­benzene­sulfonamido)­phenyl 3-methyl­benzene­sulfonate

**DOI:** 10.1107/S1600536811054808

**Published:** 2012-01-07

**Authors:** Belal O. Al-Najjar, Tengku Sifzizul Tengku Muhammad, Habibah A. Wahab, Mohd Mustaqim Rosli, Hoong-Kun Fun

**Affiliations:** a Pharmaceutical Design and Simulation (PhDS) Laboratory, School of Pharmaceutical Sciences, Universiti Sains Malaysia, 11800 Minden, Pulau Pinang, Malaysia; b Malaysian Institute of Pharmaceuticals and Nutraceuticals, Ministry of Science, Technology and Innovation, SAINS@USM, No. 10, 11900 Persiaran Bukit Jambul, Pulau Pinang, Malaysia; c Department of Biological Sciences, Universiti Malaysia Terengganu, 21030 Kuala Terengganu, Terengganu, Malaysia; d X-ray Crystallography Unit, School of Physics, Universiti Sains Malaysia, 11800 USM, Penang, Malaysia

## Abstract

The complete mol­ecule of the title compound, C_20_H_19_NO_5_S_2_, is generated by a crystallographic twofold axis and the O atom and N—H group attached to the central benzene ring are statistically disordered. The dihedral angle between the central and terminal benzene rings is 56.91 (5)° and that between the terminal benzene rings is 29.80 (5)°. In the crystal, N—H⋯O hydrogen bonding links the mol­ecules into sheets lying parallel to the *ab* plane.

## Related literature

For the biological properties of sulfonyl derivatives, see: Supuran *et al.* (2003 ▶). For a related structure, see: Sinha *et al.* (2011 ▶). For the stability of the temperature controller used in the data collection, see: Cosier & Glazer (1986 ▶).
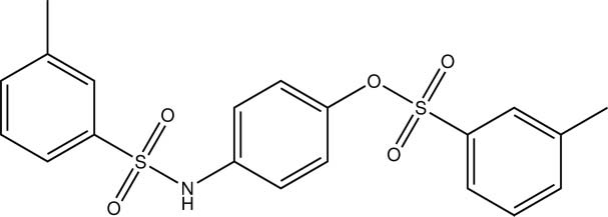


## Experimental

### 

#### Crystal data

C_20_H_19_NO_5_S_2_
*M* *_r_* = 417.48
Monoclinic, 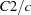

*a* = 14.4352 (1) Å
*b* = 9.1250 (1) Å
*c* = 15.4402 (2) Å
β = 109.700 (1)°
*V* = 1914.76 (4) Å^3^
*Z* = 4
Mo *K*α radiation
μ = 0.31 mm^−1^
*T* = 100 K
0.41 × 0.34 × 0.25 mm

#### Data collection

Bruker SMART APEXII CCD diffractometer
Absorption correction: multi-scan (*SADABS*; Bruker, 2009 ▶) *T*_min_ = 0.883, *T*_max_ = 0.926
21937 measured reflections
3533 independent reflections
3249 reflections with *I* > 2σ(*I*)
*R*_int_ = 0.022

#### Refinement

*R*[*F* ^2^ > 2σ(*F* ^2^)] = 0.031
*wR*(*F* ^2^) = 0.090
*S* = 1.08
3533 reflections
128 parameters
H-atom parameters constrained
Δρ_max_ = 0.46 e Å^−3^
Δρ_min_ = −0.40 e Å^−3^

### 

Data collection: *APEX2* (Bruker, 2009 ▶); cell refinement: *SAINT* (Bruker, 2009 ▶); data reduction: *SAINT*; program(s) used to solve structure: *SHELXTL* (Sheldrick, 2008 ▶); program(s) used to refine structure: *SHELXTL*; molecular graphics: *SHELXTL*; software used to prepare material for publication: *SHELXTL* and *PLATON* (Spek, 2009 ▶).

## Supplementary Material

Crystal structure: contains datablock(s) I, global. DOI: 10.1107/S1600536811054808/hb6544sup1.cif

Structure factors: contains datablock(s) I. DOI: 10.1107/S1600536811054808/hb6544Isup2.hkl

Supplementary material file. DOI: 10.1107/S1600536811054808/hb6544Isup3.cml

Additional supplementary materials:  crystallographic information; 3D view; checkCIF report

## Figures and Tables

**Table 1:** Hydrogen-bond geometry (Å, °)

*D*—H⋯*A*	*D*—H	H⋯*A*	*D*⋯*A*	*D*—H⋯*A*
N1—H1⋯O3^i^	1.02	1.97	2.9854 (11)	178

Symmetry code: (i) 
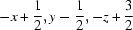
.

**Fig. 1.**
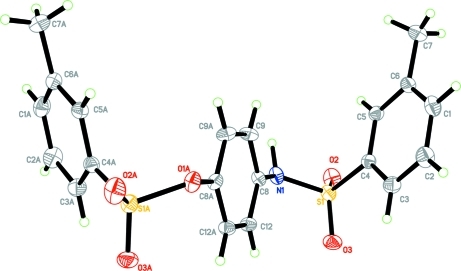
A disorder component of the structure with 50% probability displacement ellipsoids. Hydrogen atoms are shown as spheres of arbitrary radius.

**Fig. 2.**
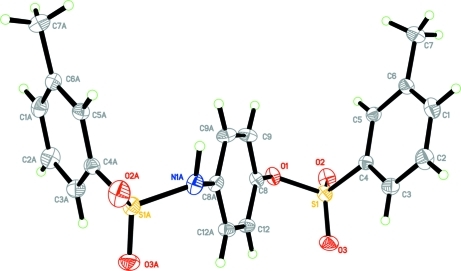
The other disorder component of the structure with 50% probability displacement ellipsoids. Hydrogen atoms are shown as spheres of arbitrary radius.

**Fig. 3.**
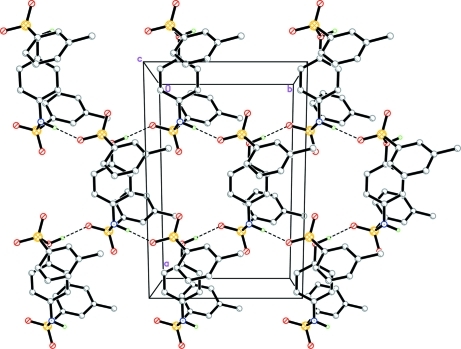
The crystal packing of (I). Dashed lines indicate hydrogen bonds. H atoms not involved in the hydrogen bond interactions have been omitted for clarity.

## supplementary crystallographic information

### Comment

Sulfonyl compounds have attracted our interest and many others, due to their varied biological activities (Sinha *et al.*, 2011). Sulfonyl derivatives are found to be active against inflammation, various viral infections as well as cancer (Supuran *et al.*, 2003).

The asymmetric unit of the title compound consists of half the molecule with the other half of the molecule being generated by a twofold axis. The crystal structure is disordered with the O1 and the N1 atoms attached at the same position with half occupancies each to the central phenyl ring (Fig 1 and Fig 2). All parameters in (I) are within normal ranges. The dihedral angle between C1/C6 and C8—C12/C8a—C12*a* is 56.91 (5)° whereas the dihedral angle between C1—C6 and C1a—C6a is 29.80 (5)°. In the crystal structure, (Fig. 3), N1—H1···O3^i^ hydrogen bonds (Table 1) link the molecules into infinite layers parallel to *ab*-plane.

### Experimental

0.02 mole of *m*-toluenesulfonyl chloride was added to 0.01 mole of *p*-aminophenol dissolved in pyridine. The reaction mixture was then neutralized by adding hydrochloric acid. The precipitate formed was dissolved in 5% aqueous sodium hydroxide and the sulfonamide recovered by adding 1:1 hydrochloric acid slowly. Re-crystallization of the product by ethanol gave colourless blocks of the title compound.

### Refinement

N bound H atom is located from a difference Fourier maps and refined using a riding model. The remaining H atoms were positioned geometrically and refined using a riding model with C—H = 0.93–0.96Å and *U*_iso_(H) = 1.2 or 1.5 *U*_eq_(*C*-methyl). A rotating group model was applied to the methyl groups. The crystal structure is disordered with N1 and O1 occupying the same phenyl position with refined site of occupancies closed to 0.5. In the final refinement, the ratio was fixed at half occupancy.

### Crystal data

**Table:**

C_20_H_19_NO_5_S_2_	*F*(000) = 872
*M**_r_* = 417.48	*D*_x_ = 1.448 Mg m^−^^3^
Monoclinic, *C*2/*c*	Mo *K*α radiation, λ = 0.71073 Å
Hall symbol: -C 2yc	Cell parameters from 9887 reflections
*a* = 14.4352 (1) Å	θ = 2.7–32.8°
*b* = 9.1250 (1) Å	µ = 0.31 mm^−^^1^
*c* = 15.4402 (2) Å	*T* = 100 K
β = 109.700 (1)°	Block, colourless
*V* = 1914.76 (4) Å^3^	0.41 × 0.34 × 0.25 mm
*Z* = 4	

### Data collection

**Table:**

Bruker SMART APEXII CCD diffractometer	3533 independent reflections
Radiation source: fine-focus sealed tube	3249 reflections with *I* > 2σ(*I*)
graphite	*R*_int_ = 0.022
φ and ω scans	θ_max_ = 33.0°, θ_min_ = 2.7°
Absorption correction: multi-scan (*SADABS*; Bruker, 2009)	*h* = −22→19
*T*_min_ = 0.883, *T*_max_ = 0.926	*k* = −13→13
21937 measured reflections	*l* = −23→23

### Refinement

**Table:**

Refinement on *F*^2^	Primary atom site location: structure-invariant direct methods
Least-squares matrix: full	Secondary atom site location: difference Fourier map
*R*[*F*^2^ > 2σ(*F*^2^)] = 0.031	Hydrogen site location: inferred from neighbouring sites
*wR*(*F*^2^) = 0.090	H-atom parameters constrained
*S* = 1.08	*w* = 1/[σ^2^(*F*_o_^2^) + (0.0485*P*)^2^ + 1.0937*P*] where *P* = (*F*_o_^2^ + 2*F*_c_^2^)/3
3533 reflections	(Δ/σ)_max_ = 0.001
128 parameters	Δρ_max_ = 0.46 e Å^−^^3^
0 restraints	Δρ_min_ = −0.40 e Å^−^^3^

### Special details

**Table:**

Experimental. The crystal was placed in the cold stream of an Oxford Cryosystems Cobra open-flow nitrogen cryostat (Cosier & Glazer, 1986) operating at 100.0 (1) K.
Geometry. All e.s.d.'s (except the e.s.d. in the dihedral angle between two l.s. planes) are estimated using the full covariance matrix. The cell e.s.d.'s are taken into account individually in the estimation of e.s.d.'s in distances, angles and torsion angles; correlations between e.s.d.'s in cell parameters are only used when they are defined by crystal symmetry. An approximate (isotropic) treatment of cell e.s.d.'s is used for estimating e.s.d.'s involving l.s. planes.
Refinement. Refinement of *F*^2^ against ALL reflections. The weighted *R*-factor *wR* and goodness of fit *S* are based on *F*^2^, conventional *R*-factors *R* are based on *F*, with *F* set to zero for negative *F*^2^. The threshold expression of *F*^2^ > σ(*F*^2^) is used only for calculating *R*-factors(gt) *etc*. and is not relevant to the choice of reflections for refinement. *R*-factors based on *F*^2^ are statistically about twice as large as those based on *F*, and *R*- factors based on ALL data will be even larger.

### Fractional atomic coordinates and isotropic or equivalent isotropic displacement parameters (Å^2^)

**Table:**

	*x*	*y*	*z*	*U*_iso_*/*U*_eq_	Occ. (<1)
S1	0.261632 (15)	0.38804 (2)	0.813408 (15)	0.01868 (7)	
O1	0.30077 (5)	0.33201 (8)	0.73257 (5)	0.01910 (13)	0.50
N1	0.30077 (5)	0.33201 (8)	0.73257 (5)	0.01910 (13)	0.50
H1	0.2719	0.2309	0.7120	0.023*	0.50
O2	0.15974 (5)	0.34770 (9)	0.78153 (5)	0.02715 (16)	
O3	0.28871 (6)	0.53930 (8)	0.83108 (6)	0.02607 (15)	
C1	0.43833 (8)	0.12376 (12)	1.06033 (7)	0.02529 (19)	
H1A	0.4772	0.0678	1.1116	0.030*	
C2	0.46090 (8)	0.27063 (12)	1.05424 (7)	0.0268 (2)	
H2A	0.5145	0.3140	1.1012	0.032*	
C3	0.40519 (7)	0.35443 (11)	0.97948 (7)	0.02235 (17)	
H3A	0.4195	0.4552	0.9750	0.027*	
C4	0.32808 (6)	0.28698 (9)	0.91157 (6)	0.01695 (15)	
C5	0.30453 (7)	0.14007 (10)	0.91733 (6)	0.01931 (16)	
H5A	0.2511	0.0969	0.8701	0.023*	
C6	0.35980 (8)	0.05647 (10)	0.99286 (7)	0.02188 (17)	
C7	0.33362 (11)	−0.10114 (11)	1.00160 (9)	0.0333 (2)	
H7A	0.3116	−0.1471	0.9407	0.050*	
H7B	0.2807	−0.1058	1.0279	0.050*	
H7C	0.3915	−0.1531	1.0419	0.050*	
C8	0.40253 (6)	0.33636 (9)	0.74375 (6)	0.01551 (14)	
C9	0.45112 (6)	0.20366 (9)	0.74745 (6)	0.01958 (16)	
H9A	0.4176	0.1138	0.7465	0.023*	
C12	0.45042 (6)	0.46948 (9)	0.74605 (6)	0.01721 (15)	
H12A	0.4160	0.5593	0.7423	0.021*	

### Atomic displacement parameters (Å^2^)

**Table:**

	*U*^11^	*U*^22^	*U*^33^	*U*^12^	*U*^13^	*U*^23^
S1	0.01452 (11)	0.02118 (11)	0.02237 (11)	0.00479 (7)	0.00888 (8)	0.00390 (7)
O1	0.0117 (3)	0.0267 (3)	0.0194 (3)	0.0003 (2)	0.0059 (2)	0.0029 (2)
N1	0.0117 (3)	0.0267 (3)	0.0194 (3)	0.0003 (2)	0.0059 (2)	0.0029 (2)
O2	0.0132 (3)	0.0395 (4)	0.0301 (4)	0.0052 (3)	0.0090 (3)	0.0047 (3)
O3	0.0290 (4)	0.0177 (3)	0.0358 (4)	0.0067 (3)	0.0166 (3)	0.0039 (3)
C1	0.0242 (4)	0.0312 (5)	0.0205 (4)	0.0031 (4)	0.0076 (3)	0.0049 (3)
C2	0.0220 (4)	0.0330 (5)	0.0220 (4)	−0.0049 (4)	0.0030 (3)	−0.0008 (4)
C3	0.0209 (4)	0.0220 (4)	0.0235 (4)	−0.0041 (3)	0.0066 (3)	−0.0019 (3)
C4	0.0160 (3)	0.0172 (3)	0.0188 (3)	0.0010 (3)	0.0074 (3)	−0.0003 (3)
C5	0.0208 (4)	0.0180 (4)	0.0201 (4)	−0.0017 (3)	0.0081 (3)	−0.0016 (3)
C6	0.0271 (4)	0.0196 (4)	0.0221 (4)	0.0013 (3)	0.0125 (3)	0.0021 (3)
C7	0.0497 (7)	0.0203 (4)	0.0344 (5)	−0.0012 (4)	0.0201 (5)	0.0050 (4)
C8	0.0119 (3)	0.0183 (3)	0.0161 (3)	−0.0001 (3)	0.0044 (3)	0.0026 (3)
C9	0.0171 (4)	0.0143 (3)	0.0236 (4)	−0.0018 (3)	0.0019 (3)	0.0025 (3)
C12	0.0149 (3)	0.0150 (3)	0.0221 (4)	0.0011 (3)	0.0068 (3)	0.0000 (3)

### Geometric parameters (Å, °)

**Table:**

S1—O2	1.4329 (8)	C4—C5	1.3931 (12)
S1—O3	1.4354 (8)	C5—C6	1.3969 (13)
S1—O1	1.6167 (7)	C5—H5A	0.9500
S1—C4	1.7572 (9)	C6—C7	1.5046 (14)
O1—C8	1.4206 (10)	C7—H7A	0.9800
O1—H1	1.0188	C7—H7B	0.9800
C1—C2	1.3899 (15)	C7—H7C	0.9800
C1—C6	1.3968 (15)	C8—C9	1.3909 (12)
C1—H1A	0.9500	C8—C12	1.3921 (12)
C2—C3	1.3922 (14)	C9—C9^i^	1.3866 (18)
C2—H2A	0.9500	C9—H9A	0.9500
C3—C4	1.3894 (13)	C12—C12^i^	1.3949 (16)
C3—H3A	0.9500	C12—H12A	0.9500
			
O2—S1—O3	119.64 (5)	C4—C5—C6	119.72 (9)
O2—S1—O1	103.82 (4)	C4—C5—H5A	120.1
O3—S1—O1	107.89 (4)	C6—C5—H5A	120.1
O2—S1—C4	111.11 (4)	C1—C6—C5	118.30 (9)
O3—S1—C4	107.83 (5)	C1—C6—C7	121.23 (9)
O1—S1—C4	105.59 (4)	C5—C6—C7	120.45 (10)
C8—O1—S1	120.90 (6)	C6—C7—H7A	109.5
C8—O1—H1	111.2	C6—C7—H7B	109.5
S1—O1—H1	108.3	H7A—C7—H7B	109.5
C2—C1—C6	121.55 (9)	C6—C7—H7C	109.5
C2—C1—H1A	119.2	H7A—C7—H7C	109.5
C6—C1—H1A	119.2	H7B—C7—H7C	109.5
C1—C2—C3	120.17 (9)	C9—C8—C12	121.29 (8)
C1—C2—H2A	119.9	C9—C8—O1	117.85 (7)
C3—C2—H2A	119.9	C12—C8—O1	120.78 (8)
C4—C3—C2	118.32 (9)	C9^i^—C9—C8	119.46 (5)
C4—C3—H3A	120.8	C9^i^—C9—H9A	120.3
C2—C3—H3A	120.8	C8—C9—H9A	120.3
C3—C4—C5	121.92 (8)	C8—C12—C12^i^	119.23 (5)
C3—C4—S1	118.94 (7)	C8—C12—H12A	120.4
C5—C4—S1	119.08 (7)	C12^i^—C12—H12A	120.4
			
O2—S1—O1—C8	−172.37 (7)	C3—C4—C5—C6	−0.44 (13)
O3—S1—O1—C8	59.70 (8)	S1—C4—C5—C6	176.91 (7)
C4—S1—O1—C8	−55.39 (7)	C2—C1—C6—C5	1.03 (15)
C6—C1—C2—C3	−0.35 (16)	C2—C1—C6—C7	−177.82 (10)
C1—C2—C3—C4	−0.71 (15)	C4—C5—C6—C1	−0.63 (14)
C2—C3—C4—C5	1.11 (14)	C4—C5—C6—C7	178.23 (9)
C2—C3—C4—S1	−176.24 (8)	S1—O1—C8—C9	113.82 (8)
O2—S1—C4—C3	−144.83 (8)	S1—O1—C8—C12	−69.45 (10)
O3—S1—C4—C3	−11.90 (9)	C12—C8—C9—C9^i^	−0.70 (16)
O1—S1—C4—C3	103.24 (8)	O1—C8—C9—C9^i^	176.01 (10)
O2—S1—C4—C5	37.75 (8)	C9—C8—C12—C12^i^	−1.20 (15)
O3—S1—C4—C5	170.68 (7)	O1—C8—C12—C12^i^	−177.81 (10)
O1—S1—C4—C5	−74.19 (8)		

Symmetry codes: (i) −*x*+1, *y*, −*z*+3/2.

### Hydrogen-bond geometry (Å, °)

**Table:**

*D*—H···*A*	*D*—H	H···*A*	*D*···*A*	*D*—H···*A*
N1—H1···O3^ii^	1.02	1.97	2.9854 (11)	178

Symmetry codes: (ii) −*x*+1/2, *y*−1/2, −*z*+3/2.

## References

[bb1] Bruker (2009). *APEX2*, *SAINT* and *SADABS* Bruker AXS Inc., Madison, Wisconsin, USA.

[bb2] Cosier, J. & Glazer, A. M. (1986). *J. Appl. Cryst.* **19**, 105–107.

[bb3] Sheldrick, G. M. (2008). *Acta Cryst.* A**64**, 112–122. 10.1107/S0108767307043930 18156677

[bb4] Sinha, S., Osman, H., Wahab, H. A., Hemamalini, M. & Fun, H.-K. (2011). *Acta Cryst.* E**67**, o3275. 10.1107/S1600536811046502 PMC3238932 22199781

[bb5] Spek, A. L. (2009). *Acta Cryst.* D**65**, 148–155. 10.1107/S090744490804362X PMC2631630 19171970

[bb6] Supuran, C. T., Casini, A. & Scozzafava, A. (2003). *Med. Res. Rev.* **23**, 535–558. 10.1002/med.10047 12789686

